# Supplementary material for: Nanoscale three-dimensional fabrication based on mechanically guided assembly
Source: Nat Commun. 2023 Feb 14;14:833. doi: 10.1038/s41467-023-36302-9 (PMC9929216; doi:10.1038/s41467-023-36302-9)
Supplement: Supplementary file 1 — Supplementary Information [file 41467_2023_36302_MOESM1_ESM.pdf]

Supplementary Information

Nanoscale Three-Dimensional Fabrication Based on Mechanically Guided Assembly

Junseong Ahn[1,2], Ji-Hwan Ha[1,2], Yongrok Jeong[1,2], Young Jung[1], Jungrak Choi[1], Jimin Gu[1], Soon Hyoung Hwang[2], Mingu Kang[1], Jiwoo Ko[1,2], Seokjoo Cho[1], Hyeonseok Han[1], Kyungnam Kang[1], Jaeho Park[1], Sohee Jeon[2], Jun-Ho Jeong[2*], Inkyu Park[1*]

[1]Department of Mechanical Engineering, Korea Advanced Institute of Science and Technology (KAIST), Daejeon 34141, Republic of Korea
E-mail: inkyu@kaist.ac.kr (I. Park); jhjeong@kimm.re.kr (J.-H. Jeong)

[2]Department of Nano Manufacturing Technology, Korea Institute of Machinery and Materials (KIMM), Daejeon 34103, Republic of Korea

Supplementary Notes 1: Summary of recently developed 3D fabrication based on mechanically guided assembly. As discussed in the main text, 3D fabrication methods based on mechanically guided assembly have been actively studied to improve design diversity with various materials[1–9] and to develop new applications[10–16] or inverse design techniques[17]. Although 3D nanostructures are in high demand[18–21], nanoscale fabrication is still challenging for most existing printing methods including mechanically guided assembly–based 3D fabrication[22,23]. Hence, we herein considered the printable size range as one of the core problems to be solved. To address this problem, we divided the 3D structures fabricated using mechanically guided assembly–based 3D fabrication into two parts, namely, bound and suspended sites, with a summary of recently reported printable sizes provided in Supplementary Fig. 1. It is worth noting that the longitudinal size of the printed structures was not considered for the following reasons. 1) For the suspended site, the length exceeds the width by one order of magnitude or more in most studies, as buckling easily occurs in high-aspect-ratio beams. 2) For the bound site, the related discussion is premature, as nanoscale alignment methods are yet to be developed, as discussed in the conclusion section. 3) Nevertheless, it is meaningful to reduce the width of the bound site as it is directly related to the design diversity and number of printable devices per unit area, and the suspended site shows width-dependent chemo-mechanical properties, as exemplified by nanowires with ultra-high surface area to volume ratios[24,25]. As macro-/microscale printing does not suffer from adhesion-related problems (Supplementary Fig. 1), the sizes of the bound and suspended sites are similar, but the relative size of the bound site increases in the case of nanoscale printing because of the weak adhesion of the substrate (Supplementary Fig. 1). In addition, the difficulty of controlling the buckling configuration of the nanoscale beam has precluded the implementation of complex structures. Therefore, we herein developed a nanoscale transfer printing method on an elastomer substrate (Fig. 2) and controlled the configuration of the nanoscale buckled beam (Fig. 3) to realize configuration-designable nanoscale 3D fabrication and thus paved the way to universal 3D nanostructure printing.

Supplementary Notes 2: Relationship between buckling configuration and mold micropatterning. First, the buckling direction (upward or downward) is determined by the direction of the initial deformation, which can be guided by the pillar edge (i.e., bound site) upon external pre-strain ($\varepsilon_{\text{substrate}}$). Second, buckling deflection is determined by the printing conditions of the applied strain on the beam, i.e., by the pre-strain of the suspended site ($\varepsilon_{\text{trench}}$). Third, buckling mode is determined by the printing conditions in terms of boundary conditions. Notably, we found that all abovementioned parameters are determined by the printing conditions in terms of the applied pre-strain and the surface microstructure of the substrate, especially the pillar width and thickness. Finite element method (FEM) simulation and experimental results show that the surface strain distribution (i.e., $\varepsilon_{\text{pillar}}$ and $\varepsilon_{\text{trench}}$) strongly depends on the pillar's aspect ratio ($t_{\text{pillar}}/w_{\text{pillar}}$) under applied pre-strain ($\varepsilon_{\text{substrate}}$), as the influence of the substrate's total strain on the pillar top decreases as the aspect ratio increases (Supplementary Fig. 9). In addition, as the aspect ratio increases, the total strain by the external strain ($\varepsilon_{\text{substrate}}$) concentrates on the strain of the trench ($\varepsilon_{\text{trench}}$), which means that the bound sites can maintain their shape in the transferred state after 3D fabrication (i.e., after the release of $\varepsilon_{\text{substrate}}$). Therefore, high aspect ratios are advantageous for the stable printing of bound sites. However, as overly high aspect ratios cause pillar bending, we used aspect ratios of 1:1 and 5:1 for the pillars. In addition, since the effect of $t_{\text{pillar}}/t_{\text{substrate}}$ ratio on the surface strain distribution was small, it was fixed at 1:50 (Supplementary Fig. 10).

Supplementary Notes 3: Electromechanical characteristics of the printed nanostructures. The electromechanical characteristics of the printed nanostructures were evaluated to fabricate rationally designed gas sensors (Fig. 5a). Upon the application of strain, the 3D nanostructure fabricated using a nanoline pattern and a pre-strain of 20% maintained its electrical resistance until an external strain of 20% (i.e., the external strain with the amount of pre-strain that was applied to the substrate) and showed negligible hysteresis, as the strain was concentrated at the suspended site (Supplementary Fig. 16). At higher external strains, the resistance drastically increased because of the fracture of nanostructures. Conversely, a gradual resistance increase was observed when external strain was applied to a nanoscale serpentine pattern on a flat elastomer substrate fabricated without pre-strain. Unlike that of a conventional microscale serpentine electrode[26], the electrical resistance of the bound nanopattern was strongly affected by substrate strain because of the low mechanical strength of the nanopattern. When a strain was applied to a 3D nanostructure fabricated using the nano-serpentine pattern and a prestrain of 20%, the initial resistance was maintained (resistance change $< 5\%$) until an external strain of 35%. In this case, the buckled 3D structures were first released within the first strain region (0–20%), and the serpentine pattern at the suspended site was then released within the second strain region (20–35%). Notably, the serpentine pattern on the bound site maintained its original shape owing to the relatively low surface strain of the pillar, as discussed in Fig. 3b, which allowed the realization of strain-insensitive electrical properties.

Supplementary Notes 4: Working mechanisms of gas sensors. First, for Pd-based H_2 gas sensor, Pd is converted to palladium hydride (PdHx) by absorbing H_2 gas under normal temperature and pressure conditions. Therefore, when Pd is exposed to H_2 gas, the electrical resistance change and mechanical deformation (volume expansion) occur because PdHx has lower electrical resistance and larger volume than pure Pd. In this study, the electrical resistance was used as a monitoring parameter to detect H_2 gas. Second, for In_2O_3-based NO_2 gas sensor, when n-type semiconductor material (i.e., In_2O_3 in this study) are thermal- or photo-activated (i.e., photo-activation in this study) and exposed to oxidizing gas (i.e., NO_2 in this study), oxygen is adsorbed on the nanostructure surface by capturing free electrons. Thus, the depletion layer of semiconductor material becomes large and electrical resistance increases. In this study, NO_2 gas concentration was monitored by measuring the electrical resistance of In_2O_3.

**Deposition layer(s) (from lower to upper layers)**	**Plasma 25 min**	**Plasma 35 min**	**Plasma 45 min**
Au (100 nm)	X	O	OO
Pt (100 nm)	X	O	O
Pd (100 nm)	X	X	O
Ag (100 nm)	O	OO	OO
In_2O_3 (100 nm)	X	X	O
Ni (100 nm)	X	X	X
Cu (100 nm)	O	O	O
Cr (100 nm)	X	X	O
Fe (100 nm)	X	X	X
TiO_2 (100 nm)	X	O	O
SiO_2 (100 nm)	X	X	X
Al_2O_3 (100 nm)	X	X	X
Au/Al_2O_3 (50 nm/50 nm)	O	O	O
Au/SiO_2 (50 nm/50 nm)	O	O	O
Au/Fe (50 nm/50 nm)	O	O	O
Au/Fe/Au (40 nm/40 nm/40 nm)	X	X	O
Au/Fe/SiO_2 (40 nm/40 nm/40 nm)	X	O	O

Supplementary Table 1: Effect of plasma treatment time on the outcome of nanotransfer printing on an elastomer substrate. X = target material was not transferred, O = target material was transferred by >80%, OO = unwanted part was co-transferred because of excessive etching (see Supplementary Fig. 5). All experiments were conducted using a nanopatterned mold with a linewidth of 800 nm.

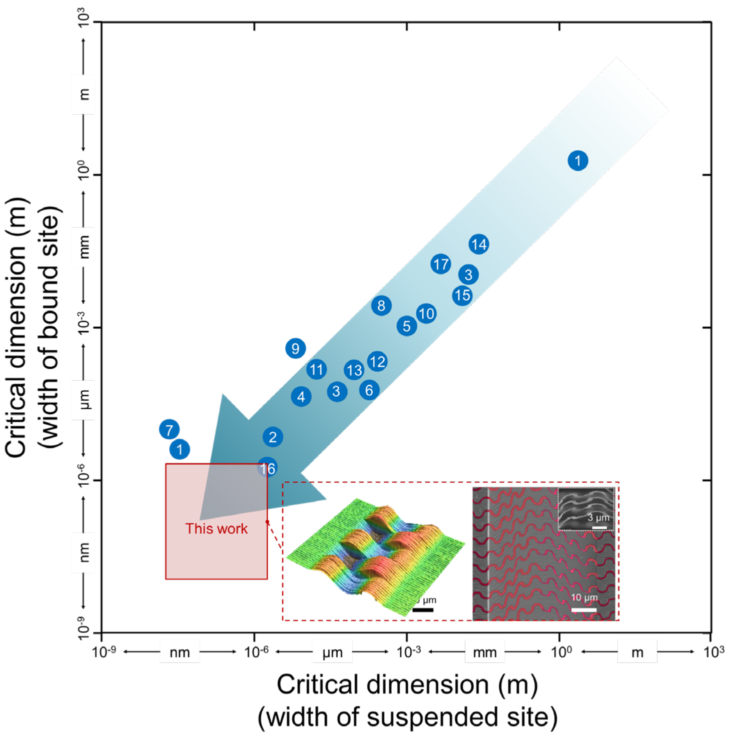

Supplementary Fig. 1: Summary of recently reported 3D fabrication methods based on mechanically guided assembly. The printed three-dimensional structures are divided into two parts (according to their functions during 3D fabrication), namely, the bound site, which is bound to the elastomer substrate to support the suspended site, and the suspended site, which is suspended and experiences buckling under the compressive strain of the substrate. In this graph, the horizontal axis is the minimum printable width of the suspended site and the vertical is the minimum printable width of the bound site.

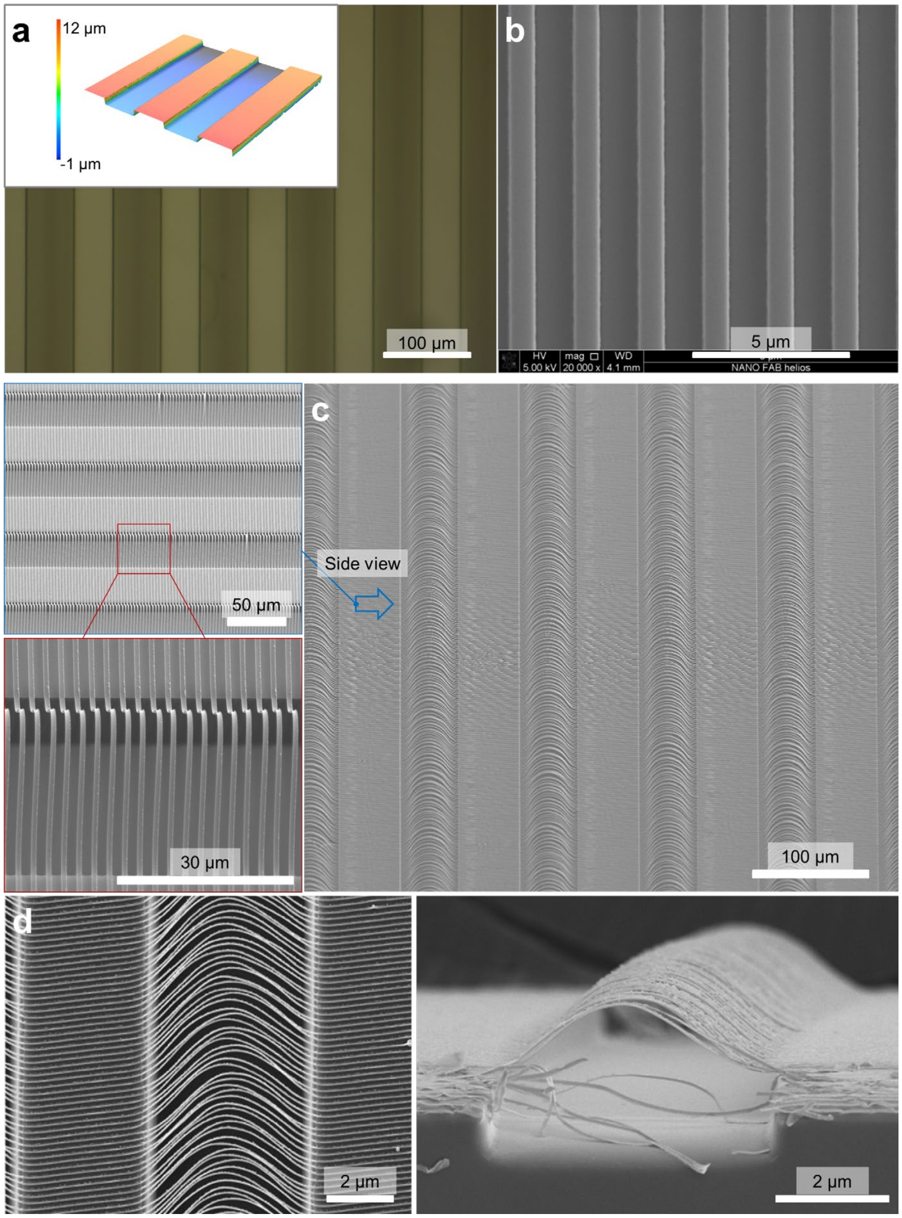

Supplementary Fig. 2: Various optical and scanning electron microscopic (SEM) images of the substrate, target material, and nanoline array. a, Confocal laser scanning microscopy and optical microscopy images of a 10-μm-thick micropatterned polydimethylsiloxane substrate. **b**, Top-view SEM image of the target material (Au) on a nanopatterned poly(urethane acrylate) mold with a linewidth of 800 nm. **c**, SEM images of a buckled nanoline array with a linewidth of 800 nm and a thickness of 100 nm acquired at various angles of view. **d**, Top- and side-view SEM images of a buckled nanoline array with a linewidth of 100 nm and a thickness of 60 nm.

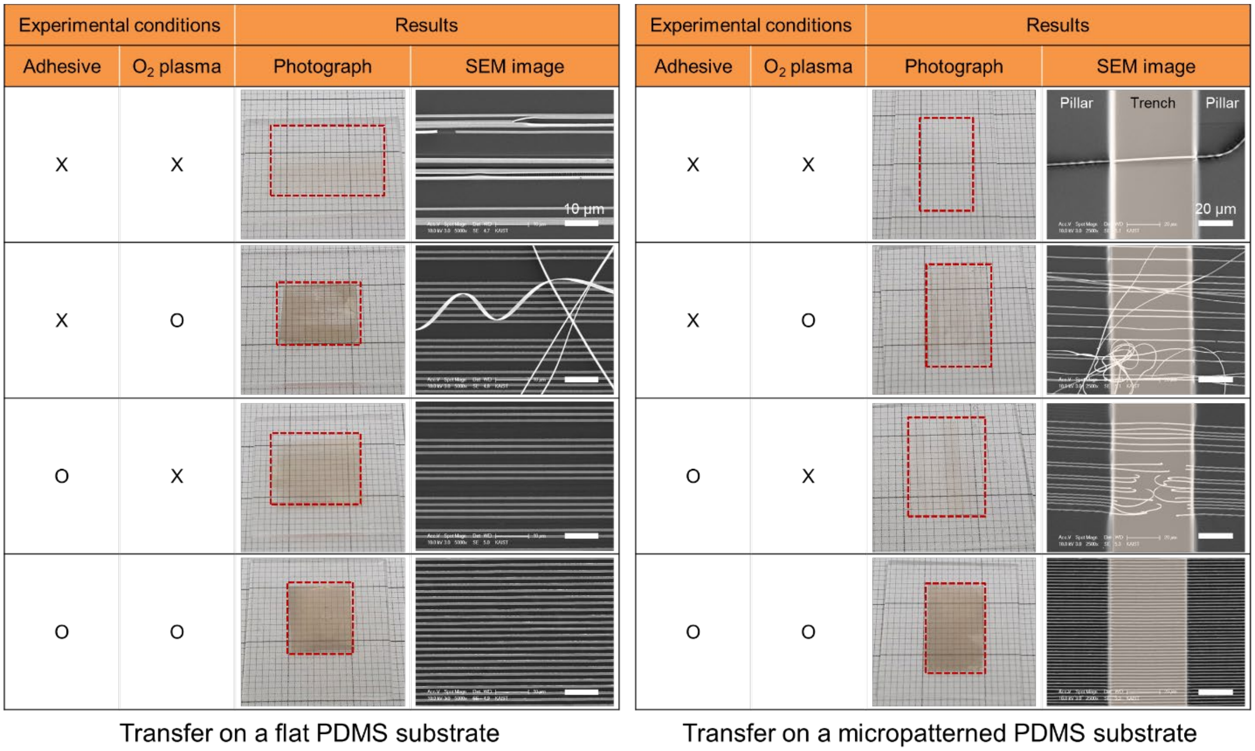

Supplementary Fig. 3: Results of two-dimensional nanotransfer printing on an elastomer substrate with/without micropatterns under different transfer conditions (i.e., with/without adhesion promoter and O_2 plasma treatment).

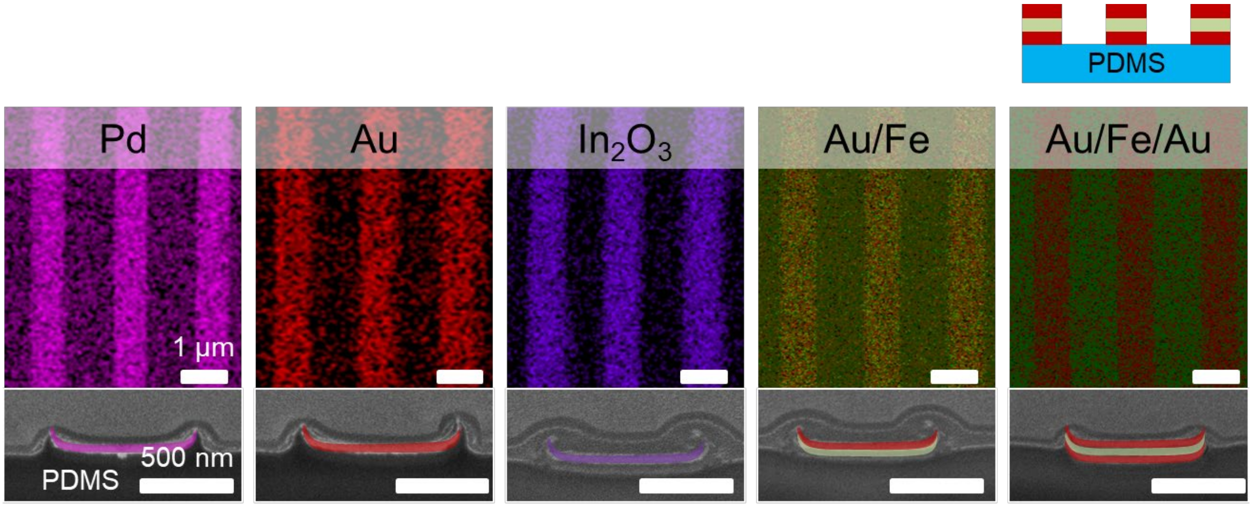

Supplementary Fig. 4: Nanotransfer printing of various materials (Pd, Au, In_2O_3, Au/Fe, and Au/Fe/Au) and multilayer structures on the elastomer substrate probed via energy-dispersive X-ray spectroscopy.

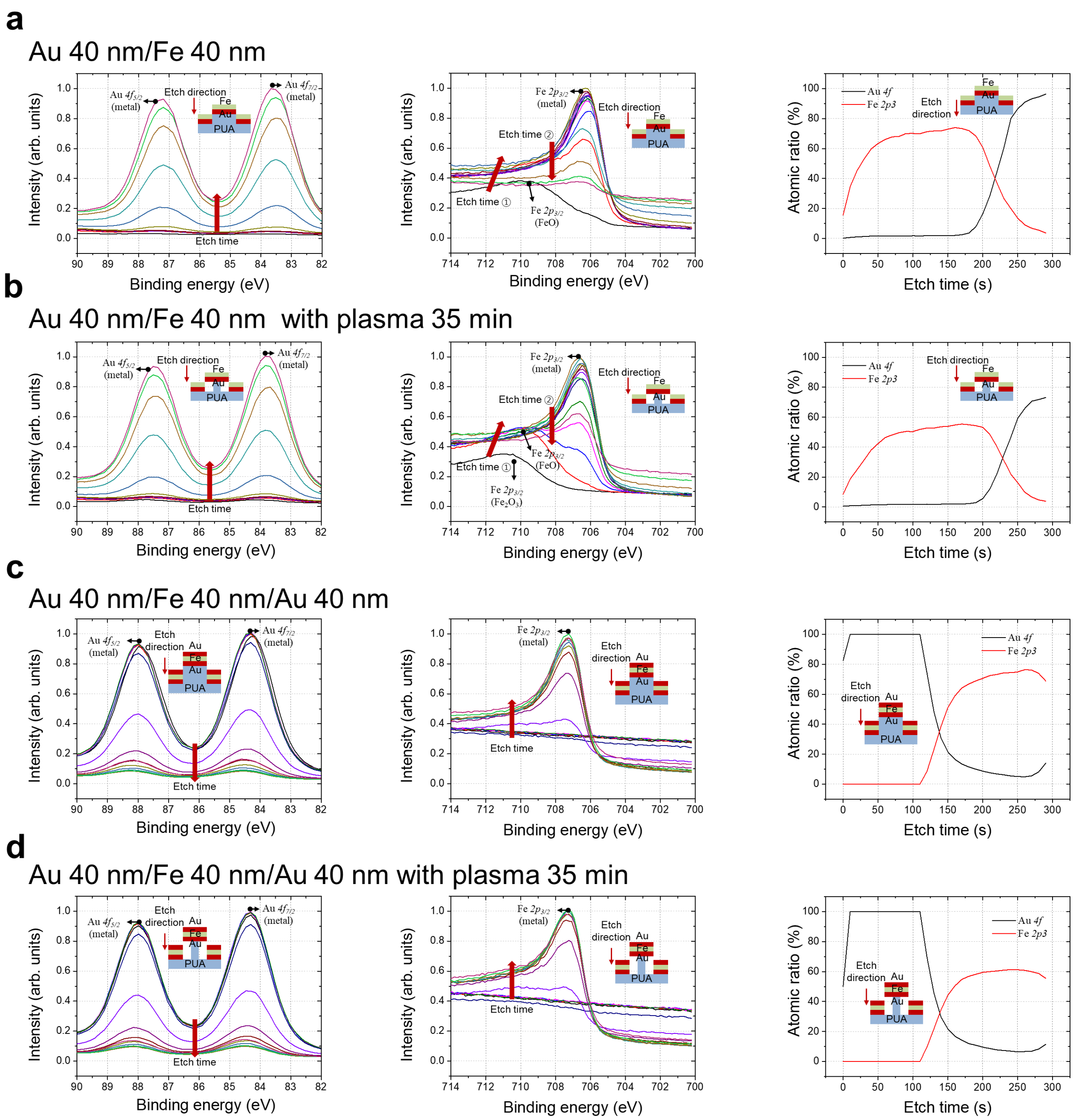

Supplementary Fig. 5: Oxidation of Fe during O_2 plasma treatment with/without the shielding layer (i.e., Au) probed via X-ray photoelectron spectroscopy. Results obtained for Au (bottom)/Fe (top) multilayer (**a**) without and (**b**) with O_2 plasma treatment and for Au (bottom)/Fe (middle)/Au (top) multilayer (**c**) without and (**d**) with O_2 plasma treatment using the nanopatterned mold. In all experiments, Au was not oxidized after deposition or O_2 plasma treatment. In the case of Fe, the surface was slightly oxidized upon direct exposure to the ambient environment and was further oxidized during plasma treatment. When encapsulated by Au, Fe was not oxidized in the ambient environment or during plasma treatment. Fe surface oxidation was examined before transfer to the elastomer substrate. Left, middle, and right

columns show the effects of etching time on the relative intensity of the Au peak, the relative intensity of the Fe peak, and the atomic ratio of the surface layer, respectively.

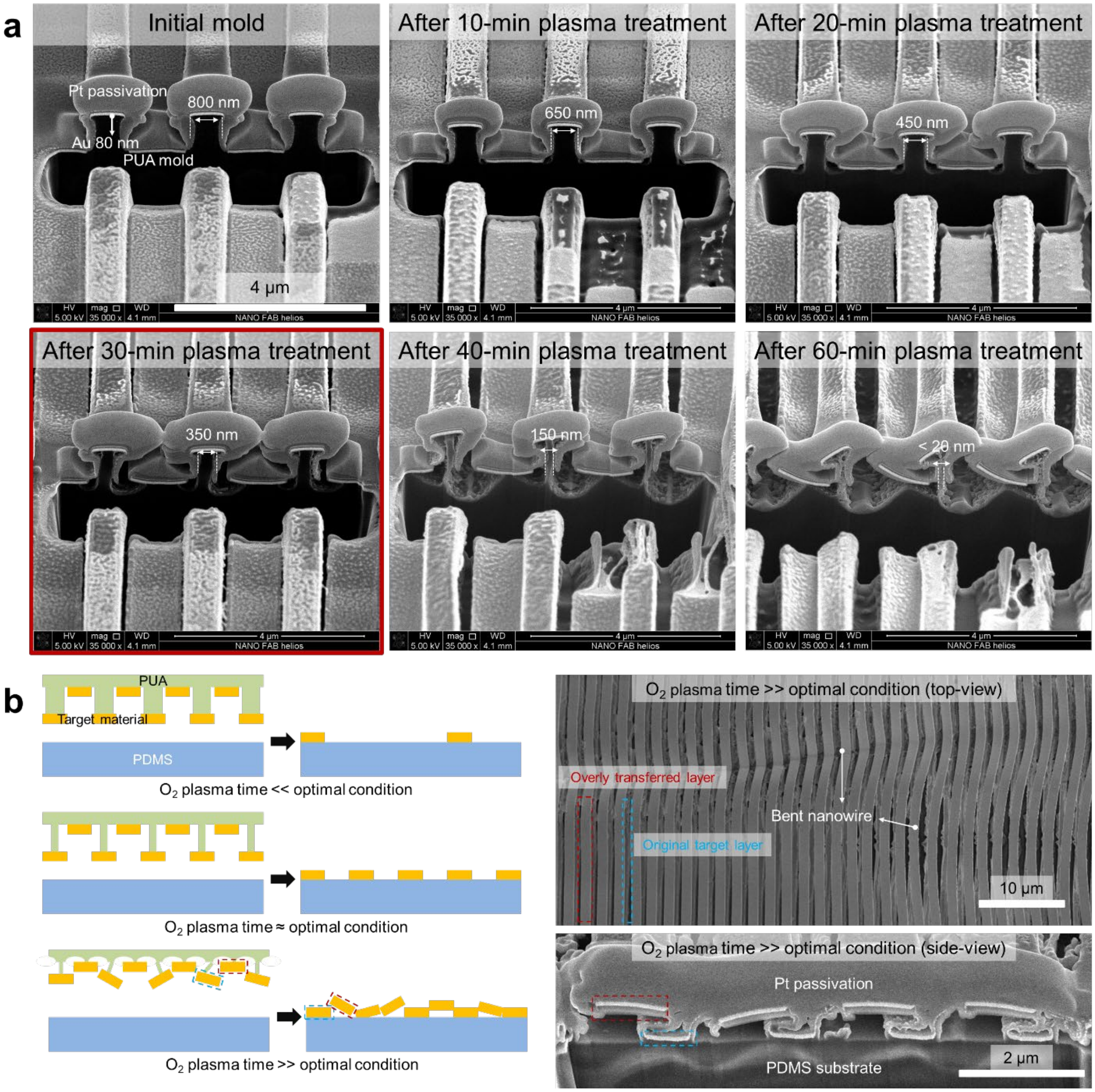

Supplementary Fig. 6: Effects of O_2 plasma treatment time probed via scanning electron microscopy. a, Side-view scanning electron microscopy images of the mold with the target material acquired for different O_2 plasma treatment times. **b**, Schematic and scanning electron microscopy image (top-view and side-view) showing the effects of plasma treatment time on the nanotransfer printing outcome.

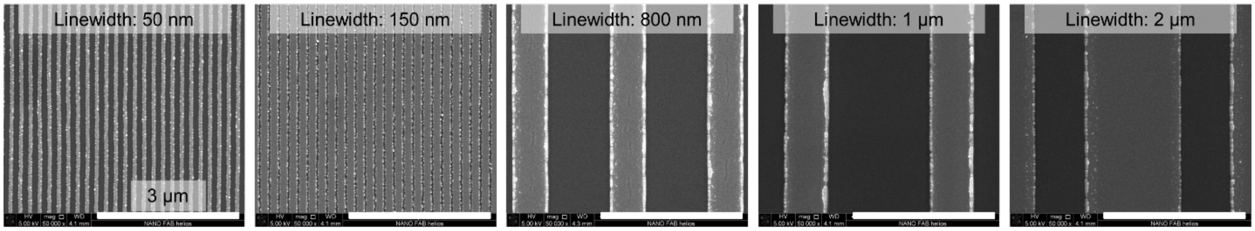

Supplementary Fig. 7: Scanning electron microscopy images showing the nanotransfer printing outcome on a flat elastomer film for different linewidths.

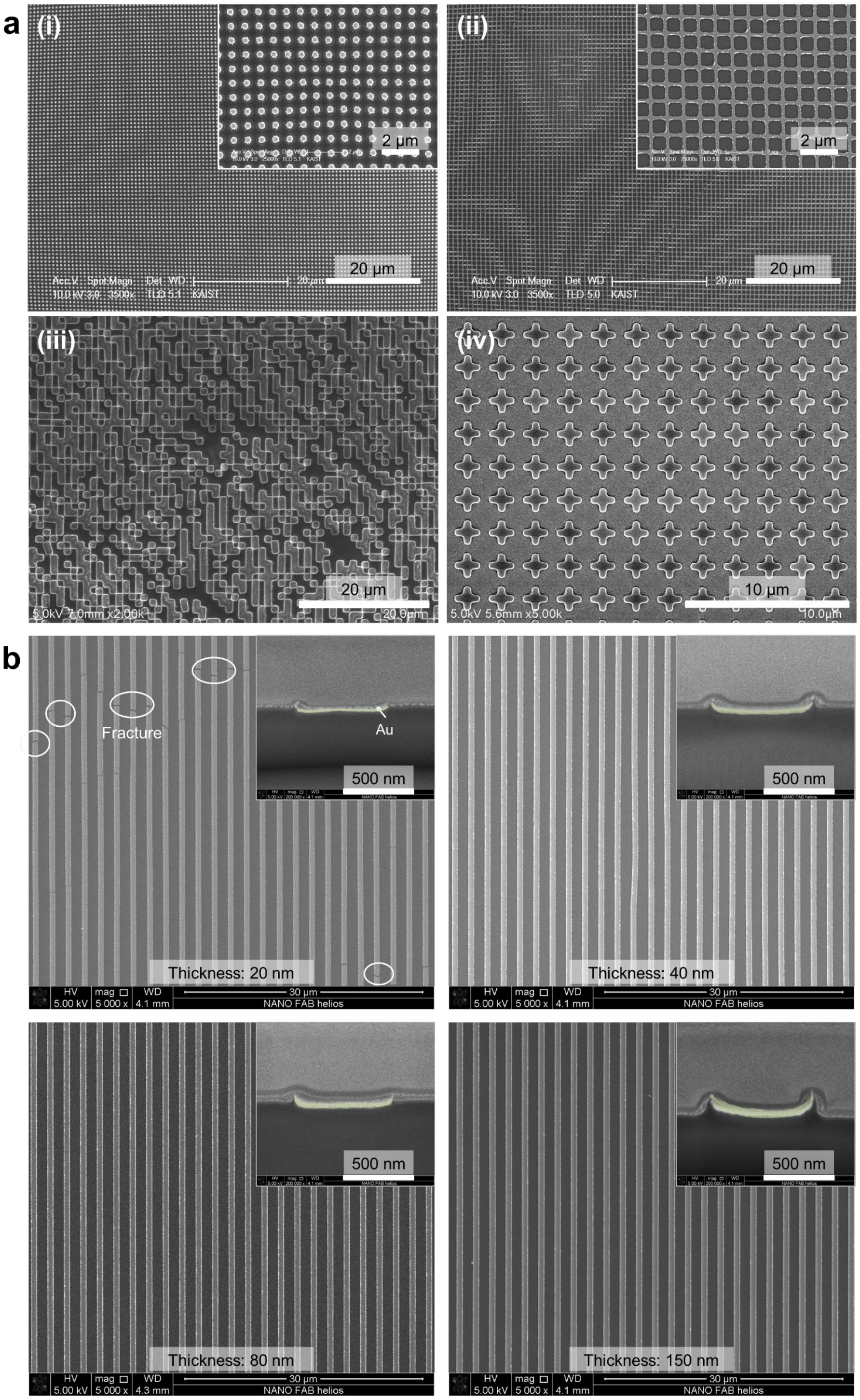

Supplementary Fig. 8: Scanning electron microscopy images showing the effects of (a) pattern shape: (i) dot, (ii) mesh, (iii) arbitrary, and (iv) cross patterns and (b) pattern thickness on the nanotransfer printing outcome. When the film thickness decreases to <20 nm, fractures are observed because of imperfect film formation and low ultimate load capacity.

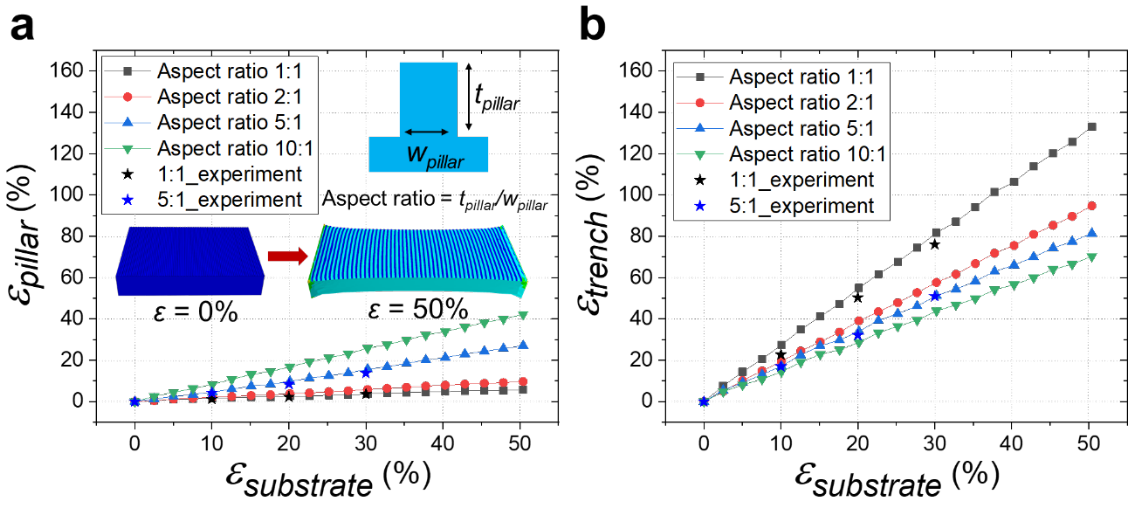

Supplementary Fig. 9: Effects of substrate micropatterning on average surface strain. Average surface strains of (**a**) the pillar (ε_{pillar}) and (**b**) trench (ε_{trench}) as functions of the external strain applied to the substrate ($\varepsilon_{substrate}$) and the aspect ratio of pillar (pillar width/pillar thickness, w_{pillar}/t_{pillar})

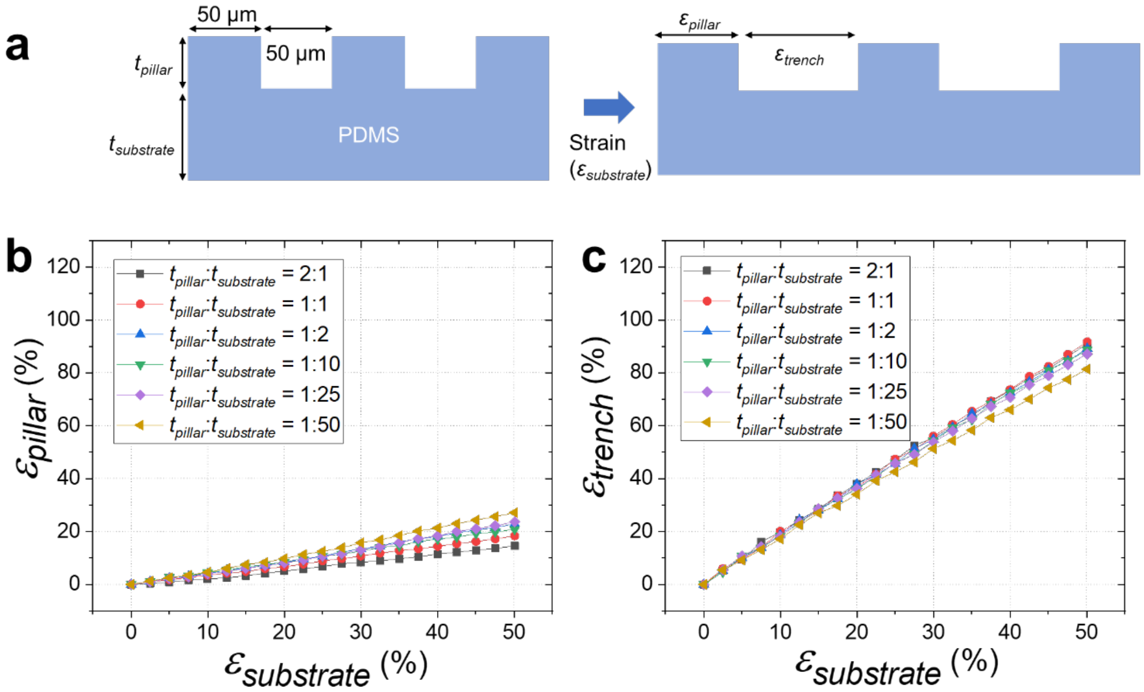

Supplementary Fig. 10: Definition of selected process parameters and their effects on the average surface strain. a, Schematic of structures with a definition of substrate design parameters. Average surface strains of (**b**) the pillar ($\varepsilon_{\text{pillar}}$) and (**c**) trench ($\varepsilon_{\text{trench}}$) as functions of the pillar thickness to substrate thickness ratio ($t_{\text{pillar}}/t_{\text{substrate}}$) and the external strain applied to the substrate ($\varepsilon_{\text{substrate}}$).

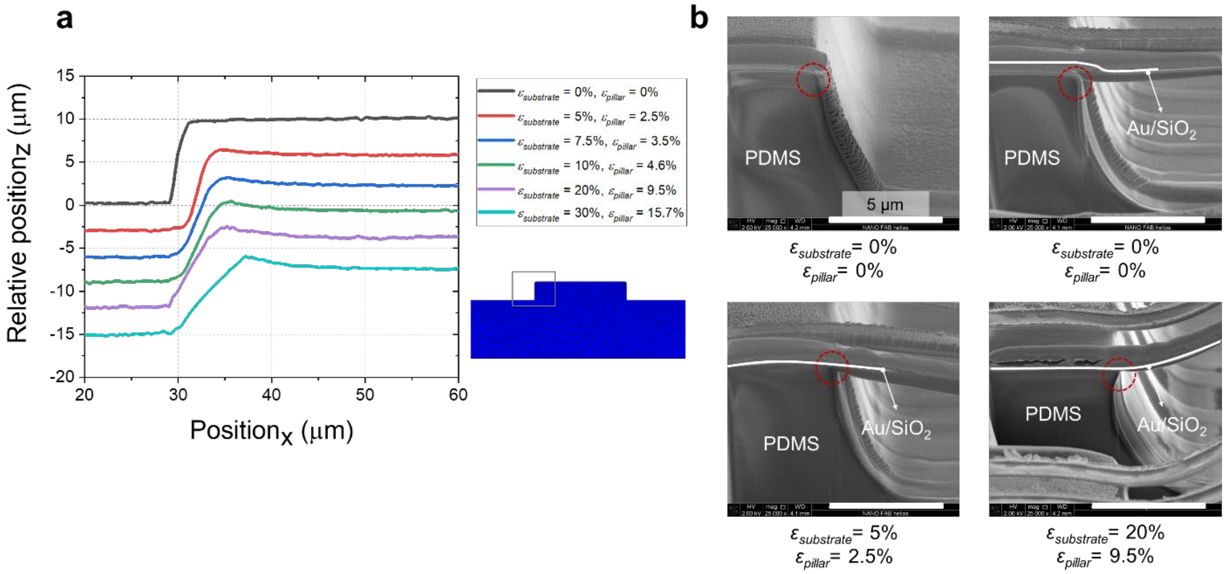

Supplementary Fig. 11: Influence of pillar strain on the pillar edge profile and buckled beam morphology. **a**, Effects of pillar strain on the pillar edge profile probed via confocal laser scanning microscopy. **b**, Scanning electron microscopy images of the pillar edge and buckled beam acquired for different initial pillar strains during pre-strain step.

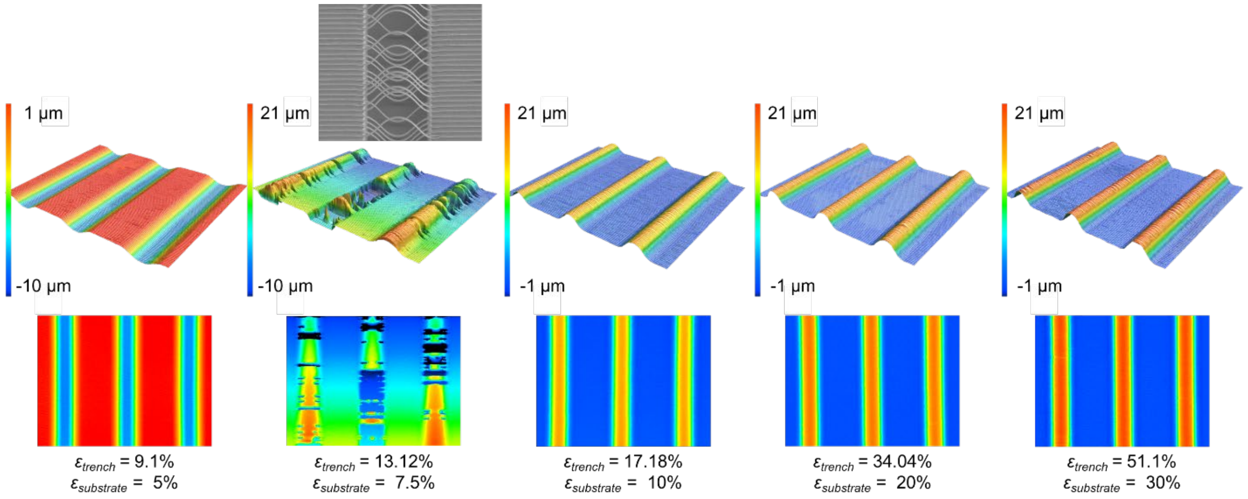

Supplementary Fig. 12: Confocal laser scanning microscopy images of buckled beam arrays acquired at different trench strains. The SEM image corresponds to that of the buckled beam array at an applied trench strain of 13.12%.

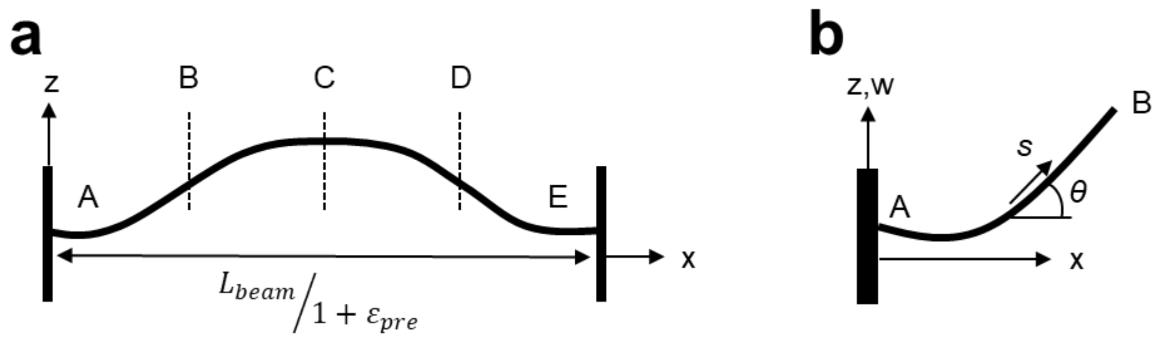

Supplementary Fig. 13: Model used to describe the buckled beam. a, Schematic of the entire buckled beam. **b**, Enlarged view showing the definition of variables used to calculate the buckling deflection. Here, L_{beam} is the length of the beam, θ is the angle which the tangent at a point of the curved beam AB makes with the x-axis, s is the distance along the axis of the curved beam from A, w is the deflection in the z-direction, and $\varepsilon_{\mathrm{pre}}$ is the amount of pre-strain applied to the substrate.

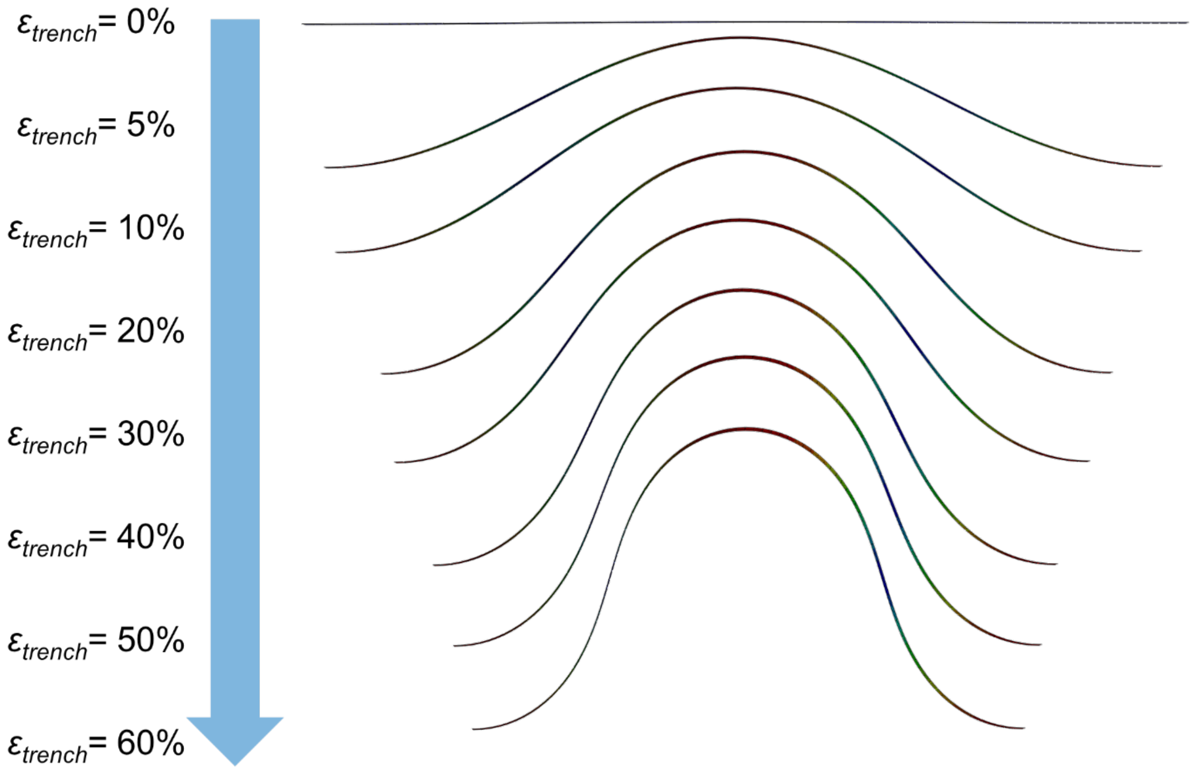

Supplementary Fig. 14: Finite element method–simulated configurations of buckled beams fabricated using different trench strains.

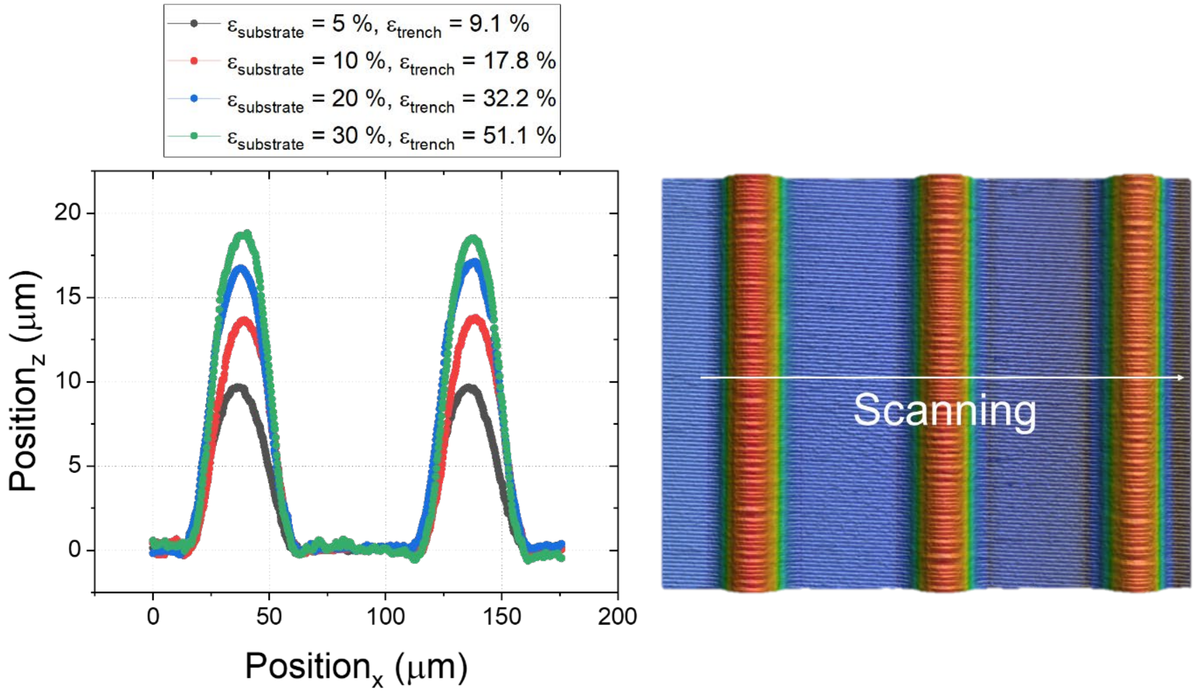

Supplementary Fig. 15: Experimentally determined configurations of beams fabricated using different substrate pre-strains ($\varepsilon_{substrate}$).

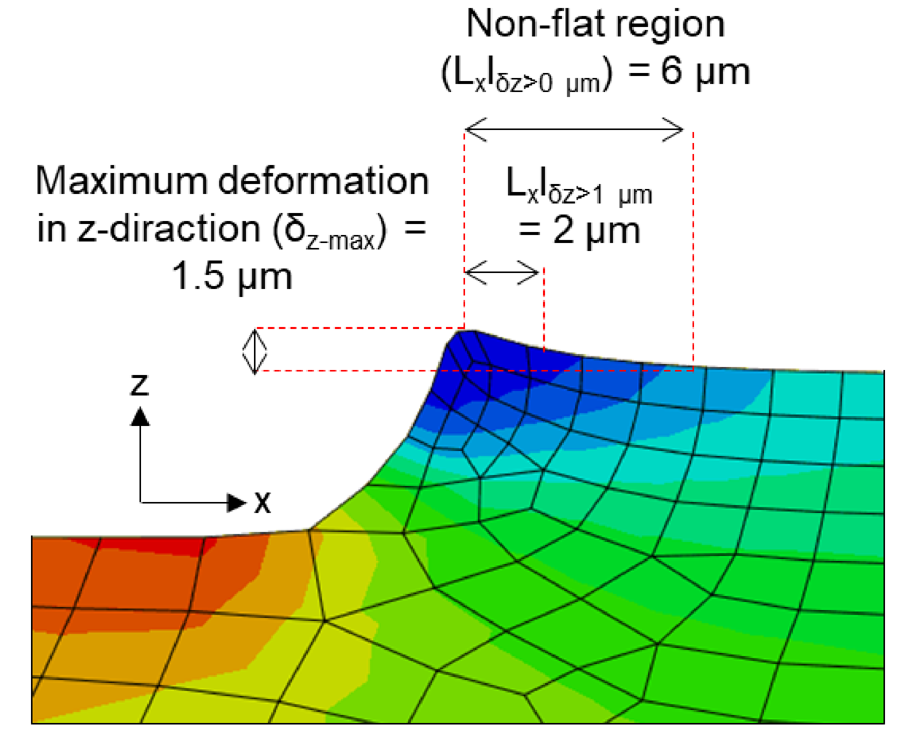

Supplementary Fig. 16: Numerical simulation for showing the amount of pillar edge deformation compared to the pillar width.

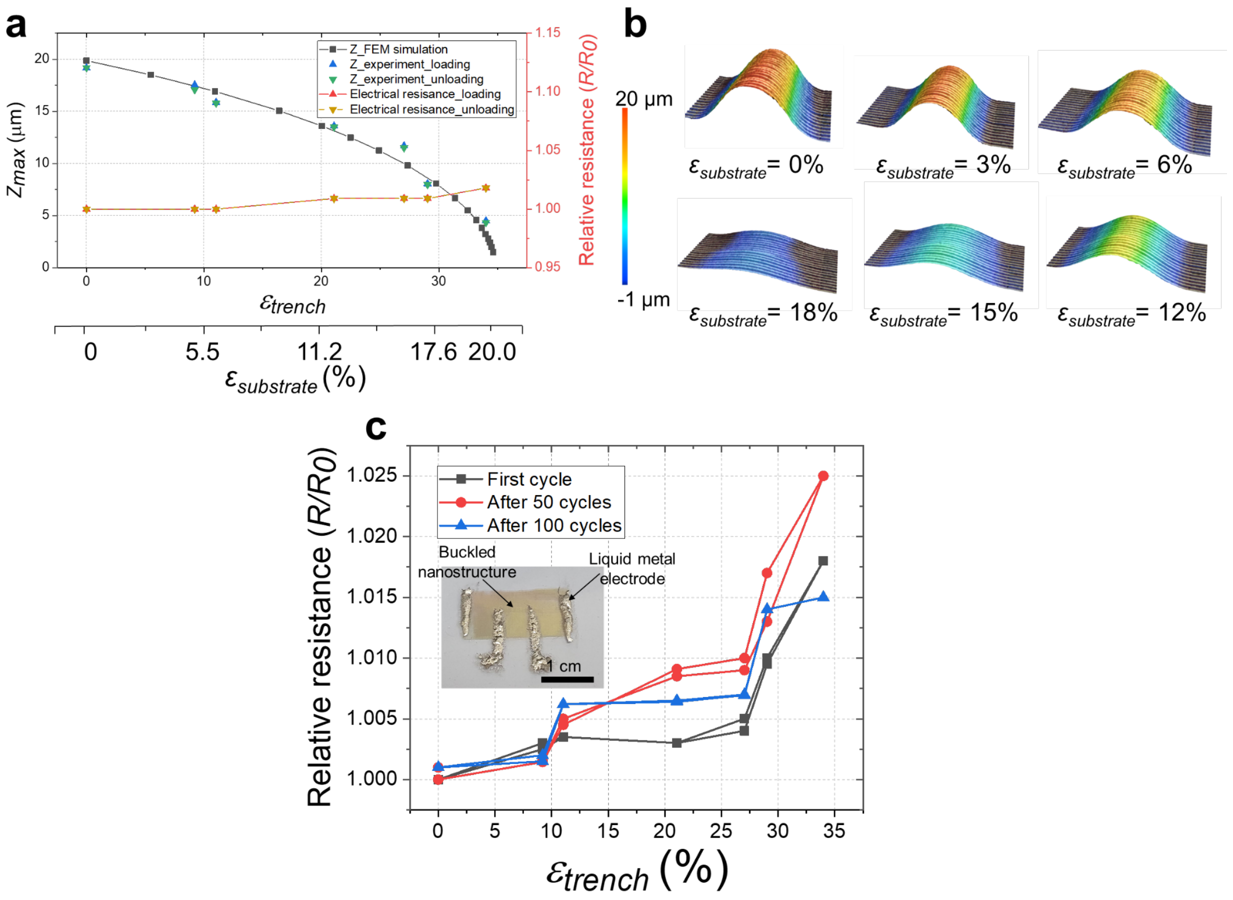

Supplementary Fig. 17: Electromechanical characterization of the buckled three-dimensional nanostructure. a, Maximum deflection and relative resistance as functions of applied strain. **b**, Confocal laser scanning microscopy images of the buckled three-dimensional nanostructure acquired at different applied strains. **c**, Relative resistance during the 100 cyclic tests with the repeated loaded/unloaded trench strain of 34% (i.e., substrate strain of 20%). As the suspended (i.e., buckled) site can absorb most of the strain, the viscoelastic behavior of the substrate did not affect the nanostructure, and no electrical or mechanical hysteresis was observed upon strain variation.

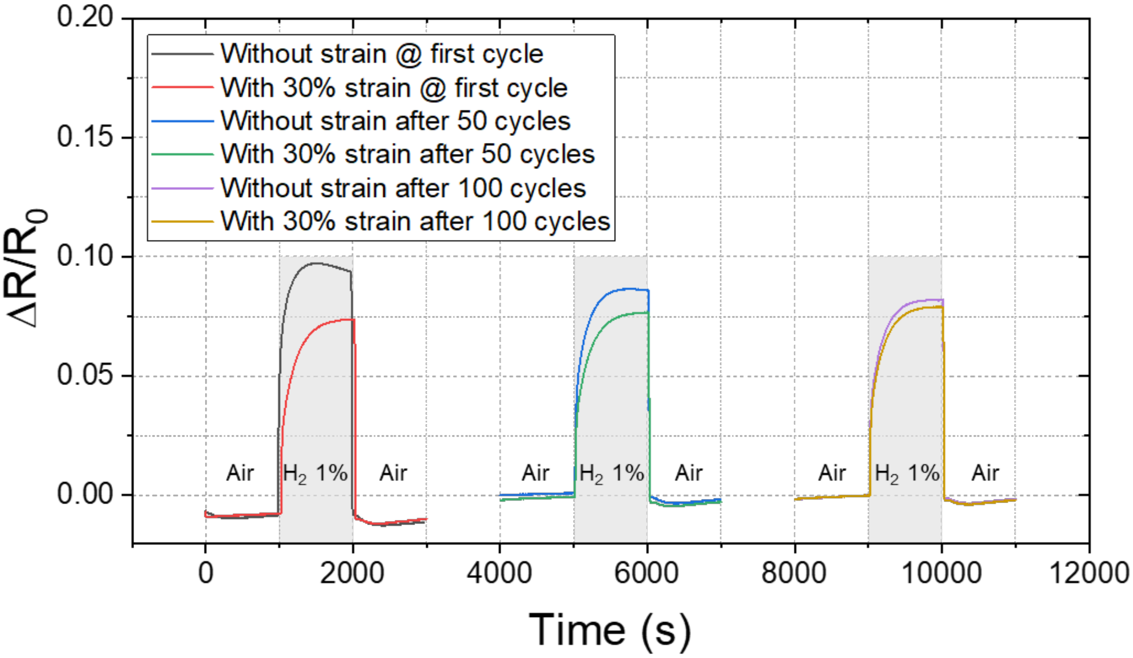

Supplementary Fig. 18: Relative resistance of a Pd nano-serpentine pattern with buckling depending on the H_2 level, external strain, and number of the repeated loaded/unloaded strain of 30%.

Supplementary References

1. Yan, Z. *et al.* Deterministic assembly of 3D mesostructures in advanced materials via compressive buckling: A short review of recent progress. *Extreme Mech Lett* **11**, 96–104 (2017).

2. Lim, S. *et al.* Assembly of Foldable 3D Microstructures Using Graphene Hinges. *Advanced Materials* **32**, 1–8 (2020).

3. Zhang, Y. *et al.* A mechanically driven form of Kirigami as a route to 3D mesostructures in micro/nanomembranes. *Proc Natl Acad Sci U S A* **112**, 11757–11764 (2015).

4. Yan, Z. *et al.* Controlled Mechanical Buckling for Origami-Inspired Construction of 3D Microstructures in Advanced Materials. *Adv Funct Mater* **26**, 2629–2639 (2016).

5. Liu, Y. *et al.* Harnessing the interface mechanics of hard films and soft substrates for 3D assembly by controlled buckling. *Proceedings of the National Academy of Sciences* **116**, 15368–15377 (2019).

6. Zhao, H. *et al.* Buckling and twisting of advanced materials into morphable 3D mesostructures. *Proc Natl Acad Sci U S A* **116**, 13239–13248 (2019).

7. Liu, W., Zou, Q., Zheng, C. & Jin, C. Metal-Assisted Transfer Strategy for Construction of 2D and 3D Nanostructures on an Elastic Substrate. *ACS Nano* **13**, 440–448 (2019).

8. Yan, Z. *et al.* Mechanical assembly of complex, 3D mesostructures from releasable multilayers of advanced materials. *Sci Adv* **2**, (2016).

9. Humood, M. *et al.* Fabrication and Deformation of 3D Multilayered Kirigami Microstructures. *Small* **14**, 1–9 (2018).

10. Kim, B. H. *et al.* Mechanically Guided Post-Assembly of 3D Electronic Systems. *Adv Funct Mater* **28**, 1–10 (2018).

11. McCracken, J. M. *et al.* Deterministic Integration of Biological and Soft Materials onto 3D Microscale Cellular Frameworks. *Adv Biosyst* **1**, 1–16 (2017).

12. Luan, H. *et al.* Complex 3D microfluidic architectures formed by mechanically guided compressive buckling. *Sci Adv* **7**, 1–13 (2021).

13. Bai, K. *et al.* Geometrically reconfigurable 3D mesostructures and electromagnetic devices through a rational bottom-up design strategy. *Sci Adv* **6**, 1–12 (2020).

14. Liu, F. *et al.* High Performance, Tunable Electrically Small Antennas through Mechanically Guided 3D Assembly. *Small* **15**, 1–9 (2019).

15. Zhao, H. *et al.* Compliant 3D frameworks instrumented with strain sensors for characterization of millimeter-scale engineered muscle tissues. *Proc Natl Acad Sci U S A* **118**, (2021).

16. Fu, H. *et al.* Morphable 3D mesostructures and microelectronic devices by multistable buckling mechanics. *Nat Mater* **17**, 268–276 (2018).

17. Fan, Z. *et al.* Inverse Design Strategies for 3D Surfaces Formed by Mechanically Guided Assembly. *Advanced Materials* **32**, 1–10 (2020).

18. Kim, J.-S. *et al*. Collectively Exhaustive Hybrid Triboelectric Nanogenerator Based on Flow-Induced Impacting-Sliding Cylinder for Ocean Energy Harvesting. *Advanced Energy Materials* **12**, 2103076 (2022).

19. Jung, Y. *et al.* Spherical Micro/Nano Hierarchical Structures for Energy and Water Harvesting Devices. *Small Methods* **2200248**, 1–11 (2022).

20. Ahn, J. *et al.* Morphology-controllable wrinkled hierarchical structure and its application to superhydrophobic triboelectric nanogenerator. *Nano Energy* **85**, 105978 (2021).

21. Ahn, J. *et al*. All-Recyclable Triboelectric Nanogenerator for Sustainable Ocean Monitoring Systems. *Advanced Energy Materials* **12,** 2201341 (2022).

22. Xu, S. *et al*. Assembly of micro/nanomaterials into complex, three-dimensional architectures by compressive buckling. *Science* **347**, 154-159 (2015).

23. Sun, Y., Choi, W. M., Jiang, H., Huang, Y. Y. & Rogers, J. A. Controlled buckling of semiconductor nanoribbons for stretchable electronics. *Nat Nanotechnol* **1**, 201–207 (2006).

24. Zhao, Z.-J. *et al.* 3D Layer-By-Layer Pd-Containing Nanocomposite Platforms for Enhancing the Performance of Hydrogen Sensors. *ACS Sens* (2020) doi:10.1021/acssensors.0c00211.

25. Yang, F., Taggart, D. K. & Penner, R. M. Fast, sensitive hydrogen gas detection using single palladium nanowires that resist fracture. *Nano Lett* **9**, 2177–2182 (2009).

26. Keum, H., Mccormick, M., Liu, P., Zhang, Y. & Omenetto, F. G. RESEARCH ARTICLES Epidermal Electronics. **333**, (2011).
